# Endoscopic appearances of gastric mucosa in different endoscopic models according to H. pylori infection status

**DOI:** 10.1002/jgh3.70028

**Published:** 2024-09-21

**Authors:** Shinwari Abdullah Jan, Hashimi Sayed Zekria

**Affiliations:** ^1^ Faculty of Medicine Nangarhar University Jalalabad Afghanistan

**Keywords:** conventional white light endoscopy, endoscopic appearance, endoscopic models, H. pylori infection status, image‐enhanced endoscopy, Kyoto classification

## Abstract

**Background:**

H. pylori infection has been recognized as a type 1 carcinogen of the gastric malignancy; therefore, early diagnosis and treatment are the corner stone of eradication. Recent findings have also shown that atrophy and intestinal metaplasia remain after successful eradication, which moderately increases the risk of gastric cancer compared with those who have never infected, so the evaluation of gastric mucosa during gastroscopy is important.

**Aims:**

We aimed to describe and summarize the reliable literature and proposed features of H. pylori infection status and gastritis in research on newly developed endoscopic models that influence clinical practice. In the result, conventional white light endoscopic, image‐enhanced endoscopic models, and studies related to the Kyoto classification of gastritis were searched and reviewed.

**Results:**

Kyoto classification of gastritis and modified Kyoto classification scoring model for gastritis using conventional white light image (CWLI) endoscopy is an effective tool for evaluating current H. pylori infection status, past infections, eradications, noninfections, and pre‐cancerous conditions. This model is widely used, low cost, and time‐efficient, and is supported by recent findings. Advanced image‐enhanced endoscopic models combined with magnifying endoscopy provide more clear endoscopic features for H. pylori infection status and early gastric cancer.

**Conclusion:**

According to H pylori infection status, endoscopic prediction of gastric mucosal surface architecture analysis is possible, which influences clinical management. Endoscopic models might lead us to accurate and early diagnose of H. pylori infection status and may not be effective only for the eradication of H. pylori infection but also in the detection of early gastric cancer status.

## Graphical abstract

This study highlights the endoscopic appearance of gastric mucosa using various models, including Conventional White Light Imaging (CWLI), Image‐Enhanced Endoscopy (IEE), and the Kyoto classification system for gastritis. These models are effective in distinguishing H. pylori infection status, including current, eradicated, non‐infected, and pre‐cancerous conditions. The use of WLI technique, due to their global accessibility, low cost, and time efficiency, aids in accurate diagnosis and early detection of H. pylori‐related diseases and gastric cancer.

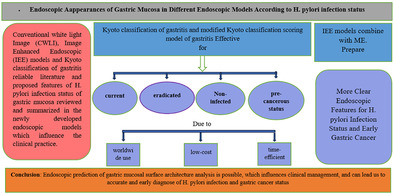

## Introduction

Newly developed models of endoscopic examination can detect the atrophy and intestinal metaplasia of the gastric mucosa that may persist after eradication of H. pylori infection and increase the risk of gastric cancer relative to those never infected. Accordingly, during endoscopy, evaluation of the gastric mucosa for H. pylori infection, along with other diagnostic tests such as urea breathe (UBT), stool antigen test (SAT) and rapid urease test (RUT), is important not only for the diagnosis of H. pylori but also for pre‐cancerous gastric lesions. H. pylori infection prevalence is around 50% worldwide and is the leading cause of chronic gastritis,^1^ which is classified as class I carcinogen by World Health Organization and International Agency for Research of cancer.^2^ Gastric cancer is a fatal disease, with only one in five people surviving for more than 5 years.^1^ Most of gastric adenocarcinomas, particularly of intestinal type, is associated with phenotypic changes that result from chronic inflammation induced or initiated by H. pylori infection.^3^

In addition to earlier whit light image (WLI) endoscopy, several new advanced endoscopic imaging modalities and techniques have been developed for differential diagnosis of gastric lesions. In current clinical practice, magnifying endoscopy (ME) and image‐enhanced endoscopy (IEE) are essential and useful tools for detecting and accurately describing lesions with histologic features.^4^

In conventional white light image endoscopy, present gastric mucosal features are presented superficially and grossly.^5^ However, image‐enhanced endoscopy (IEE), magnifying endoscopy (ME), narrow‐band imaging (NBI), blue laser imaging (BLI), artificial intelligence (AI) and autofluorescence imaging (AFI) visualize mucosal membrane and vascular structures along with the diagnosis of H. pylori status and neoplastic lesions well than conventional white light endoscopy.^6^, ^7^

The objective of this review is to gather and summarize the gastric mucosal imaging features in both conventional white light image and IEE models to the status of H. pylori infection and identify the relevance in clinical practice. Therefore, conventional white light image, IEE models, and related studies to the Kyoto classification were searched and reviewed.

## Endoscopic features of H. pylori infection status in various endoscopic models and clinical applicability of these techniques

Nowadays, different endoscopic models and technique are used and each model can be implemented for the detection of H. pylori infection status (current or positive, eradicated, and never infected forms) by their specific endoscopic features.

### Conventional white light image endoscopic model in clinical practice

**Figure 1:**
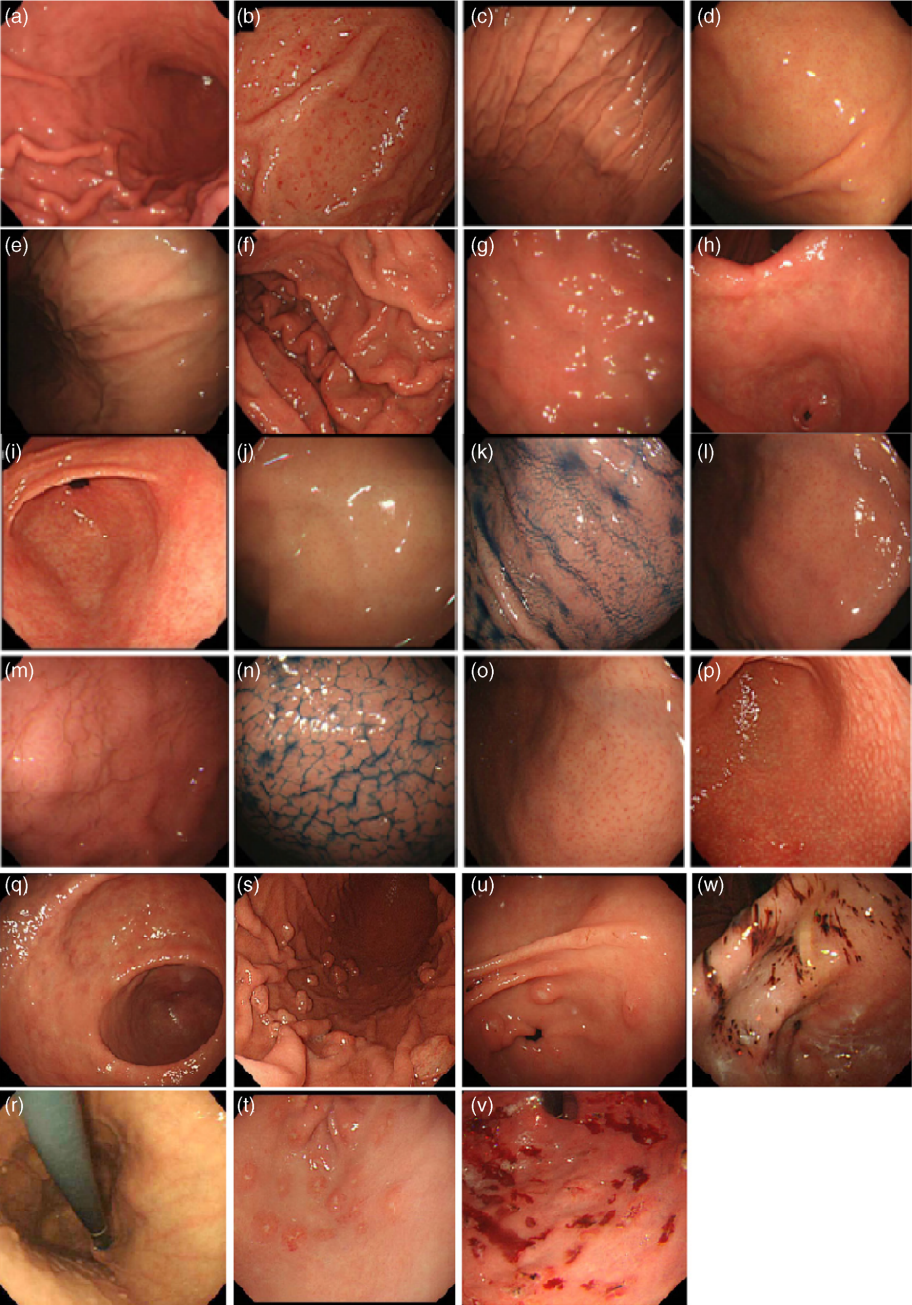
Evaluated endoscopic features of Kyoto classification of gastritis in the conventional white light image endoscopy: (a) Diffuse redness; present. (b) Diffuse redness: absent. (c) Spotty redness. (d) Fold enlargement: present. (e) Fold enlargement: absent. (f) Mucosal swelling in fundic mucosa: present. (g) Mucosa swelling in fundic mucosa: absent. (h) Mucosal swelling in pyloric mucosa: present. (i) Mucosal swelling in pyloric mucosa: absent. (j) Swelling of areae gastricae (conventional endoscopy): present. (k) Swelling of areae gastricae (conventional endoscopy): absent. (l) Swelling of areae gastricae (indigocarmine [IC] method): present. (m) Swelling of areae gastricae (IC method): absent. (n) Regular arrangement of collecting venules: present. (o) Regular arrangement of collecting venules: absent. (p) Nodular change. (q) Patchy redness. (r) Red streak. (s) Fundic gland polyposis. (t) Flat erosion. (u) Raised erosion. (v) Hemorrhagic erosion. (w) Bleeding spot.^5^, ^8^

**Figure 2:**
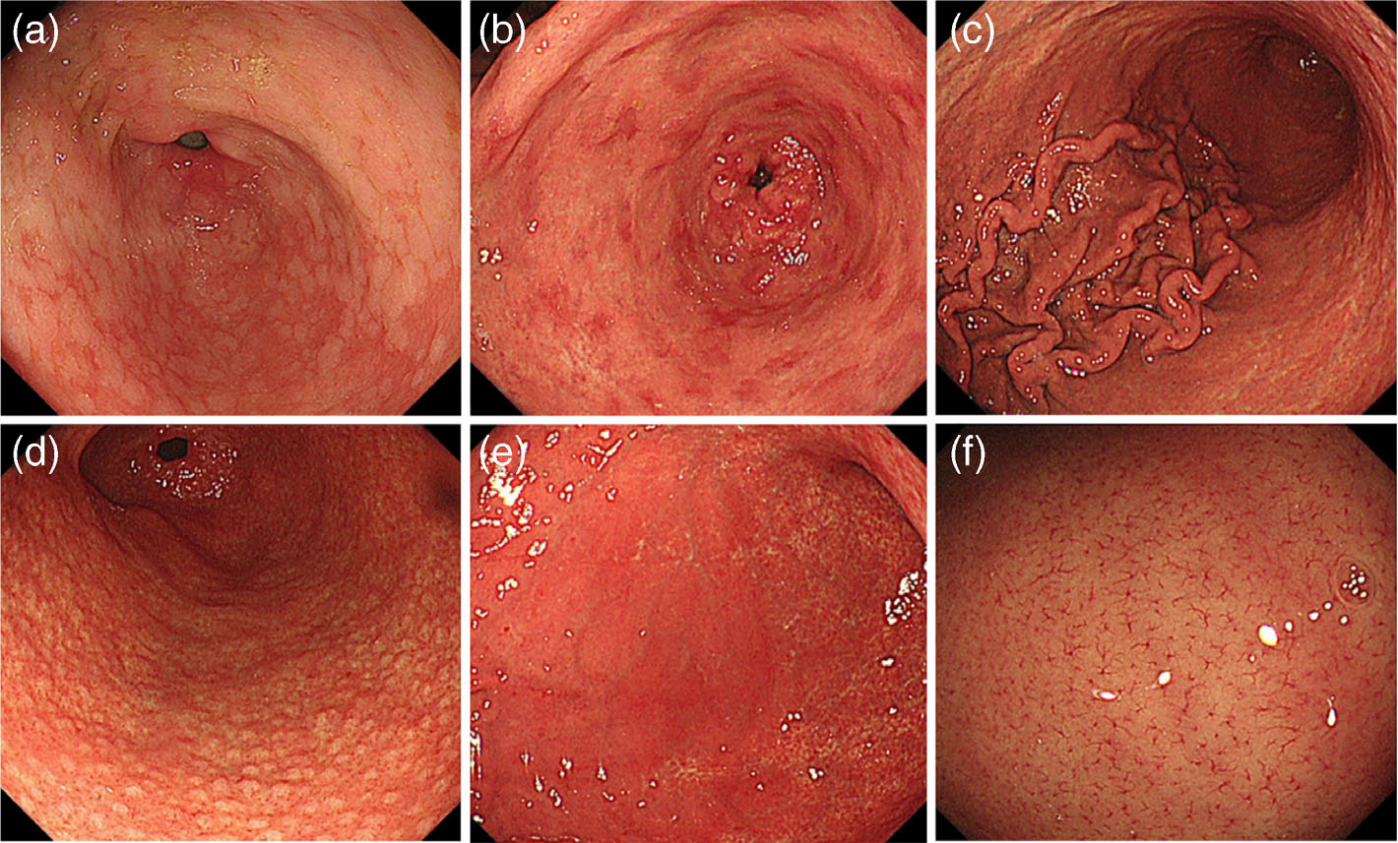
Endoscopic findings of Kyoto classification of gastritis. (a): Intestinal metaplasia; (b): Map‐like redness; (c): Enlarged folds; (d): Nodularity; (e): Diffuse redness; (f): Regular arrangement of collecting venules in weakly magnified image.^5^, ^8^

In 2013, Japan Gastroenterology Endoscopy Society developed a series of 19 conventional white light image endoscopic findings of chronic gastritis in the title of “Kyoto classification of gastritis” for the evaluation of H. pylori infection status and the risk of gastric cancer. In this classification, sticky mucus, diffuse redness, spotty redness, mucosal swelling, enlarged/tortuous fold, nodularity, xanthoma, and hyperplastic polyp predict current H. pylori positive infection; normal mucosal appearance of the antrum, angle and body, presence of regular arrangement of collecting venules (RAC), fundic gland polyp (FGP), red streak, and hematin demonstrate absence or H. pylori noninfected gastric mucosa; regression of spotty redness indicates eradicated status of H. pylori infection (see Fig. 1); mucosal atrophy, intestinal metaplasia, enlarged folds, and nodularity suggest pre‐cancerous status of gastric mucosa (see Fig. 2).^5^, ^8^ These findings are also confirmed by Yoshii et al.^9^ First, Kimura–Takemoto classification was introduced in Japan for atrophic gastritis.^10^ Moreover, gastric mucosal swelling and redness were also proposed for H. pylori‐induced inflammation.^11^

Diffuse redness like mucosal swelling is a key finding in the current H. pylori positive infection, which is clearly related to the degree of neutrophils and mononuclear cells infiltration caused by H. pylori infection.^12^, ^13^ H. pylori‐infected inflamed mucosa is characterized by continuous mucosal breakdown and vascular remodeling,^14^, ^15^ such as persistent inflammation decrease the density of irregular small vessels and regular arrangement of collecting venules (RAC) may reappear in the atrophic mucosa. However, these findings are not common for previous or past infection. H. pylori induced atrophy refers to the current infection,^16^ which gradually disappears after eradication, but usually persists even though its boundaries are unclear.^17^ Therefore, individually atrophy is not a unique feature for current or eradicated H. pylori infection. In some previous studies, raised erosion and multiple white and smooth elevated lesions are predicted for previous infection.^18^

The Kyoto classification of gastritis and other studies have proposed a characteristic feature for H. pylori non‐infected gastric mucosa, ideally observed in the gastric body, especially in the lower part of the lesser curvature, through close endoscopic examination using conventional white light image endoscopy. This was first reported by Yagi et al. in 2005.^19^ Sensitivity and specificity of the RAC appearance are 100% and 90% respectively. Nevertheless, specific view of RAC can be found for H. pylori non‐infected status in gastric antrum with conventional white light image endoscopy,^20^ but magnifying endoscopy technique can observe easily.^20^

Moreover, in 2010, H. pylori‐infected gastric mucosa evaluated by Sheng‐Lei Yan et al. by conventional white light image endoscopic and divided gastric mucosa to the H. pylori infection status into four types (see Table 1), and found that conventional white light image endoscopy could detect H. pylori infection in the gastric mucosa without atrophy.^21^

**Table 1:** H. pylori‐infected gastric mucosal types in conventional whit light image endoscopic model^21^

Type	Definition of gastric mucosal appearances	Predicting H. pylori infection status in gastric mucosa			
Type‐1	Cleft‐like appearance	1 and 2 Type patterns are statistically significant in predicting H. pylori negative status.			
Type‐2	Regular arrangement of red dots				
Type‐3	Mosaic mucosal pattern	3 and 4 Types patterns are statistically significant in predicting a H. pylori positive status.			
Type‐4	Mosaic pattern with a focal area of hyperemia				
		Sensitivity	Specificity	PPV	NPV
		100%	86%	94%	100%

Recently, the Kyoto classification of gastritis has been revised by Wang et al. and established a modified Kyoto classification scoring model that consist of five endoscopic findings and totally have eight points. In this modified scoring model, gastric mucosa atrophy, intestinal metaplasia, enlarged folds, and nodularity endoscopic features are viewed to evaluate the gastric cancer risk and H. pylori infection status. Red color mucosa and regular arrangement of collecting venules features demonstrate H. pylori infection status. Zero score predicts absence of H. pylori infection, 2 or higher score suggests H. pylori infection, and 4 or higher score predicts the risk of developing gastric cancer (see Table 2).^22^ The prevalence of H. pylori infection of modified Kyoto classification gastritis scores in the 0, 1, and ≥2 point groups are 1.5%, 45%, and 82%, respectively. Two newly defined patterns, unclear atrophic boundaries (UABs) and regular arrangement of collecting venule features, have been added to the modified Kyoto classification model of gastritis, to prepare evidence to the clinical accuracy of H. pylori gastritis and strengthen the Kyoto classification of gastritis. They assessed the H. pylori infection in three statuses: current, past or eradicated, and noninfected status. Three findings, unclear atrophic boundary, map‐like redness, and reappearance of regular arrangement of collecting venules, predict the past or eradicated infection, if one of these three findings is present, confirms the past or eradicated H. pylori infection; nodularity, diffuse redness, and mucosal swelling suggest the current positive infection and have a high ROC/AUC (0.726); and the presence of regular arrangement of collecting venules is identified as highly specific for the noninfected status.^23^

**Table 2:** Modified Kyoto classification scoring model of gastritis

Endoscopic findings	Scores	Descriptions
Atrophy		
None, C1	0	C1 = Atrophy is limited to the antrum.
C2 and C3	1	C2 = Atrophy is limited to the minor area of the lesser curvature of the body. C3 = Atrophy exists in the major area of the lesser curvature of the body but does not extend beyond the cardia.
O1–O3	2	O1 = Atrophy extends to the fundus over the cardia. Atrophic border of the body lies between the lesser curvature and anterior wall. O2 = Atrophic border of the body lies on the anterior wall. O3 = Atrophy is widespread with the border between the anterior wall and greater curvature.
Intestinal metaplasia		
None	0	
Confined to the antrum	1	
Extending to the gastric body	2	
Hypertrophy of the gastric fold		
Absence (<5 mm)	0	
Presence (≥5 mm)	1	
Nodularity		
Absence	0	
Presence	1	
Diffuse redness		
None	0	
Mild (RAC can be seen in gastric body)	1	
Severe (RAC disappeared in gastric body)	2	
Total modified Kyoto score	0–8	

RAC, Regular arrangement of collecting venules.

Glover et al. in 2021 strongly proposed gastric body distal part RAC appearance for the H. pylori noninfected status; diffuse redness and mucosal swelling to the current positive infection; and map‐like redness for the eradicated or past H. pylori infection status.^24^ Hirai et al. in 2023 described that careful observation of mucosal atrophy and swelling in mildly atrophic mucosa, which may conform the diagnosis. Kaplan–Meier analysis also identified a higher gastric cancer detection rate in the current positive and eradicated H. pylori status than noninfected status.^25^ Consistent with previous studies, regular arrangement of collecting venules for H. pylori noninfected status (OR = 32) and diffuse redness (OR = 26.8) for H. pylori current infection are the prominent endoscopic features, and overall diagnostic accuracy rate in the modified Kyoto classification scoring model is 82.9%.^8^ Sensitivity, specificity, and odd ratio of White light image endoscopic gastric mucosal patterns to the H. pylori status are summarized in Table 3, and a comparison with other endoscopic models are described in Tables 6, 7, 8, and Figure 7.

**Table 3:** Summary of Sensitivity, specificity, and odd ratio of white light image endoscopic gastric mucosal findings to the H. pylori infection status

		Odds ratio	Sensitivity	Specificity			
Glover et al.^26^	Mucosal edema	18.1	63.7%	91.1%	Predictor of H. pylori positive infection		
	Diffuse redness	14.4	66.5%	89.0%			
	Map‐like redness		99.0%	13.0%	Predictor of H. pylori infection Eradication		
	RAC	55.0	78.3%	93.8%	Predictor of H. pylori no infection		
Zhao et al.^23^	Nodularity	11.7	Combined feature 69.1%	Combined feature 82.5%	Predictor of H. pylori positive infection		
	Diffuse redness	10.5					
	Mucosal swelling	ROC/AUC (0.726)					
	Map‐like redness	7.78	Combined feature 37.8%	Combined feature 90.7%	Predictor of H. pylori infection Eradication		
	Unclear atrophy boundary (UAB)	7.69	Combined feature 37.8%	Combined feature 90.7%			
	Reappearance of RAC						
	Hematin	Combined feature					
	Red streak		94.3%	99.7%	Predictor of H. pylori no infection		
	RAC						
Glover et al.^24^	Diffuse redness				Predictor of H. pylori positive infection		
	Mucosal swelling						
	Map‐like redness				Predictor of H. pylori infection Eradication		
	RAC		78.4%	64.3	Predictor of H. pylori no infection		
Takuma et al.^27^	Over all gastric mucosal abnormal lesion's				PPV	NPV	Accuracy
			93.3%	89.1%	92.3%	90.6%	91.6%

In the conclusion, newly described endoscopic gastric mucosal patterns with previous endoscopic findings of conventional white light image endoscopy can be implemented in the detection of H. pylori infection status and gastric pre‐cancerous lesion as accurate and in real‐time practice.^6^

### Image‐enhanced endoscopic models in clinical practice

Magnifying endoscopy (ME), narrow‐band imaging (NBI), linked color imaging (LCI), blue laser imaging (BLI), and autofluorescence imaging (AFI) are known as IEE models that enhance the observation of gastric mucosa and mucosal surface vascular structure and have higher accuracy and detection rate in the H. pylori‐infected and pre‐cancerous status of the gastric mucosa than conventional white light endoscopic model.^6^ Advanced endoscopic imaging, especially magnifying model, improves the observation of gastric mucus membrane. Mucosal swelling in H. pylori‐associated gastritis caused by neutrophils and mononuclear cells infiltration. Vascular destruction and increasing irregular vessels cause gastric pits enlargement or elongation and inflammation disappear the collecting venules.^38^

### Magnifying endoscopy (ME)

**Figure 3:**
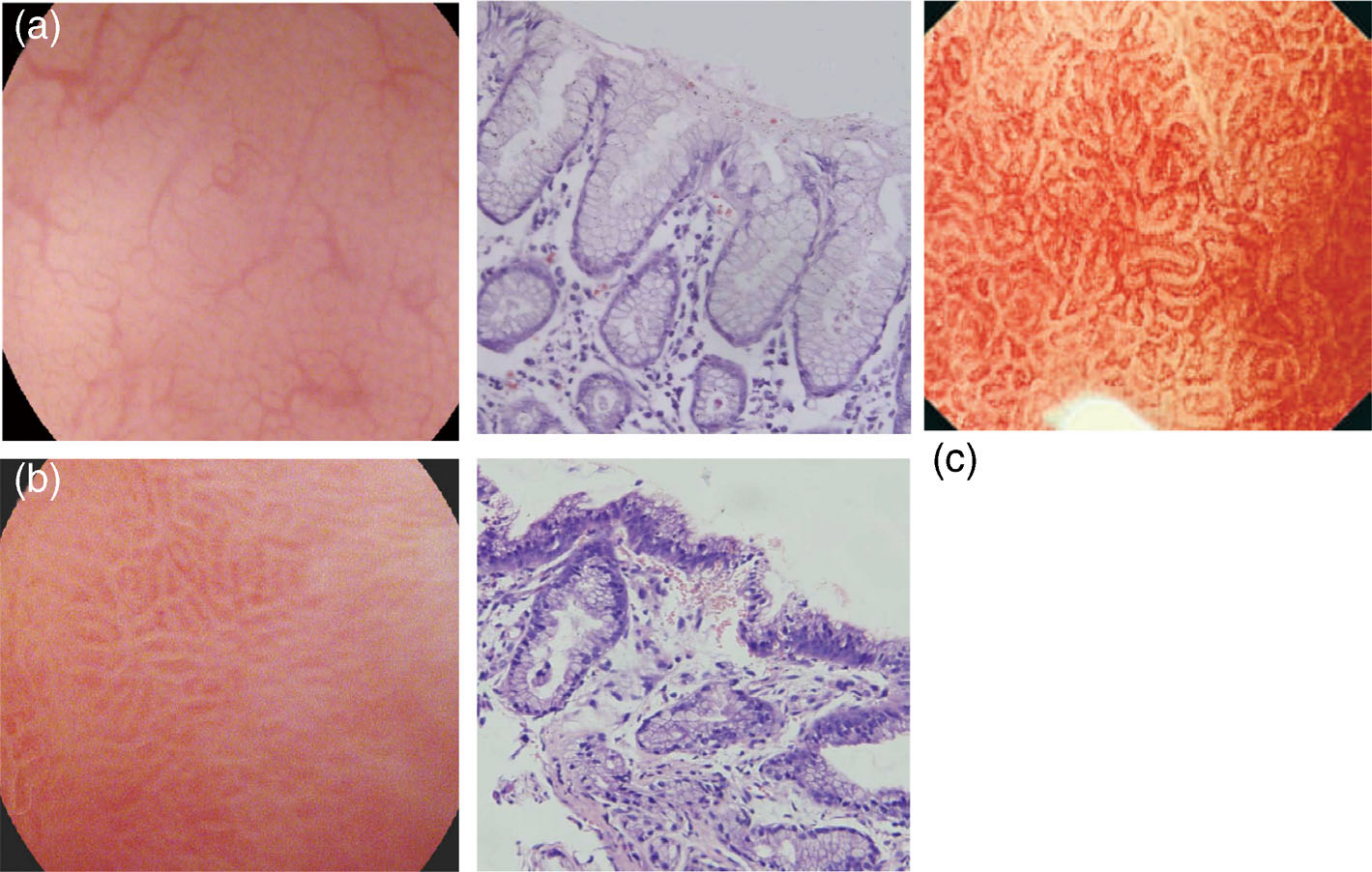
Gastric body mucosal patterns on magnifying endoscopy. (a): Normal mucosa comprises a honeycomb‐type subepithelial capillary network (SECN) with a regular arrangement of collecting venules (arrow) and regular, round pits; (b): Helicobacter pylori (H. pylori)‐associated gastritis appears loss of the normal SECN and collecting venules, with enlarged white pits surrounded by erythema; (c): Corresponding histologic features of Figure 1a; (d): Corresponding histologic features of Figure 1b.^39^ (c): In normal antral mucosa, the SECN is coil−shaped and no collecting venules can be seen.^40^

The magnifying endoscopic model enlarges the imaging area, allowing for a clear observation of the gastric mucosal membrane structures. Most magnifying endoscopes have a resolution of less than 10 μm, and during real‐time endoscopy, allow for gastric mucosal observation at 80–120 times magnification. As a result, the membrane's micro‐surface structure and the microvascular architecture surrounding the gastric pits can be observed in detail.^15^ With zoomed or magnifying endoscopic technique, the normal view of the fundal mucosal glands and vascular architectures are obviously visible in the pit patterns, mucosal glands have fixed, rounded, or oval (mouth‐like) crypt openings, which are observe as pin‐like dark spots in the central part of mucosal glands, surrounded by subepithelial capillary networks (SECNs) and made a honeycomb‐like appearance (Fig. 3).^7^ Magnifying endoscopy strengths the correlation between H. pylori‐associated gastritis and histological findings, in which a good interobserver agreement has been reported.^15^ H. pylori infection causes consistent or unchangeable chronic inflammation of gastric mucosa and infiltrated cells, degenerated epithelium, and disrupted microvascular system, causing disappearance obvious features of collecting venules (see Fig. 3).^41^

**Figure 4:**
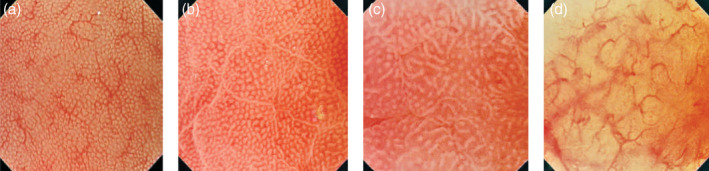
The gastric body mucosal patterns identified on magnifying endoscopy. (a): The type 1 pattern comprises a honeycomb‐type subepithelial capillary network (SECN) with a regular arrangement of collecting venules and regular, (b): The type 2 pattern comprises a honeycomb−type SECN with regular, round pits, but with loss of collecting venules, (c): In the type 3 pattern, there is loss of the normal SECN and collecting venules, with enlarged white pits surrounded by erythema. (d): The type 4 pattern is characterized by loss of the normal SECN and round pits, with irregular arrangement of the collecting venules.^40^

Yagi et al. developed a classification for the assessment of stomach mucosal membrane in magnifying endoscopy. Accordingly, Z0 demonstrates normal mucosal collecting venules and true capillaries, which surround the pit as a network and have 93.8% sensitivity and 96.2% specificity. Other three types, Z1, Z2, and Z3, indicates H. pylori‐infected mucosa.^41^ In this manner, Nakagawa's classification divides the gastric mucosa into three patterns through the RAC morphology: R = regular, I = irregular, and O = obscured patterns. R pattern illustrates H. pylori non‐infected status with 63.9% and 100% sensitivity, specificity respectively.^42^ Kawamura et al. focused on the crypts opening white mucosal membrane and divided on white pit and dens white pit. White and dense white pit predict H. pylori infection that have 81.7% and 78.5% sensitivity and specificity, respectively.^43^ Anagnostopoulos et al. divided gastric mucosa by magnifying endoscopy on four types in Western population (see Table 4 and Fig. 4) and described that the high‐resolution magnifying endoscopy is better and high model for the diagnosis of H. pylori‐related gastritis than conventional white light endoscopy.^40^

**Table 4:** Types of gastric mucosa in Western population in the magnifying endoscopy

Type	Appearances	Sensitivity	Specificity
Type 1	Regular arrangement of collecting venules and regular, round pits		
Type 2	Regular, round pits, but loss of collecting venules	For predicting H. pylori infection in type 2 and 3	
Type 3	Loss of normal collecting venules, with enlarged white pits surrounded by erythema	100%	92.7%
Type 4	Loss of normal round pits, with irregular arrangement of collecting venules	90%	96%
		Corresponded to atrophic gastritis	

### Magnifying with narrow‐band imaging endoscopy (ME‐NBI)

**Figure 5:**
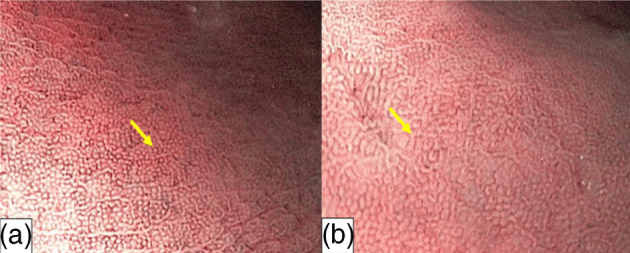
(a) H. pylori‐negative gastric mucosa is characterized by homogeneous, round pits with regular honeycomb‐like SECNs in NBI; (b) H. pylori‐positive gastric mucosa is characterized by enlarged or elongated, varies in sized and shaped of pits with unclear SECNs in NBI.^6^

**Figure 6:**
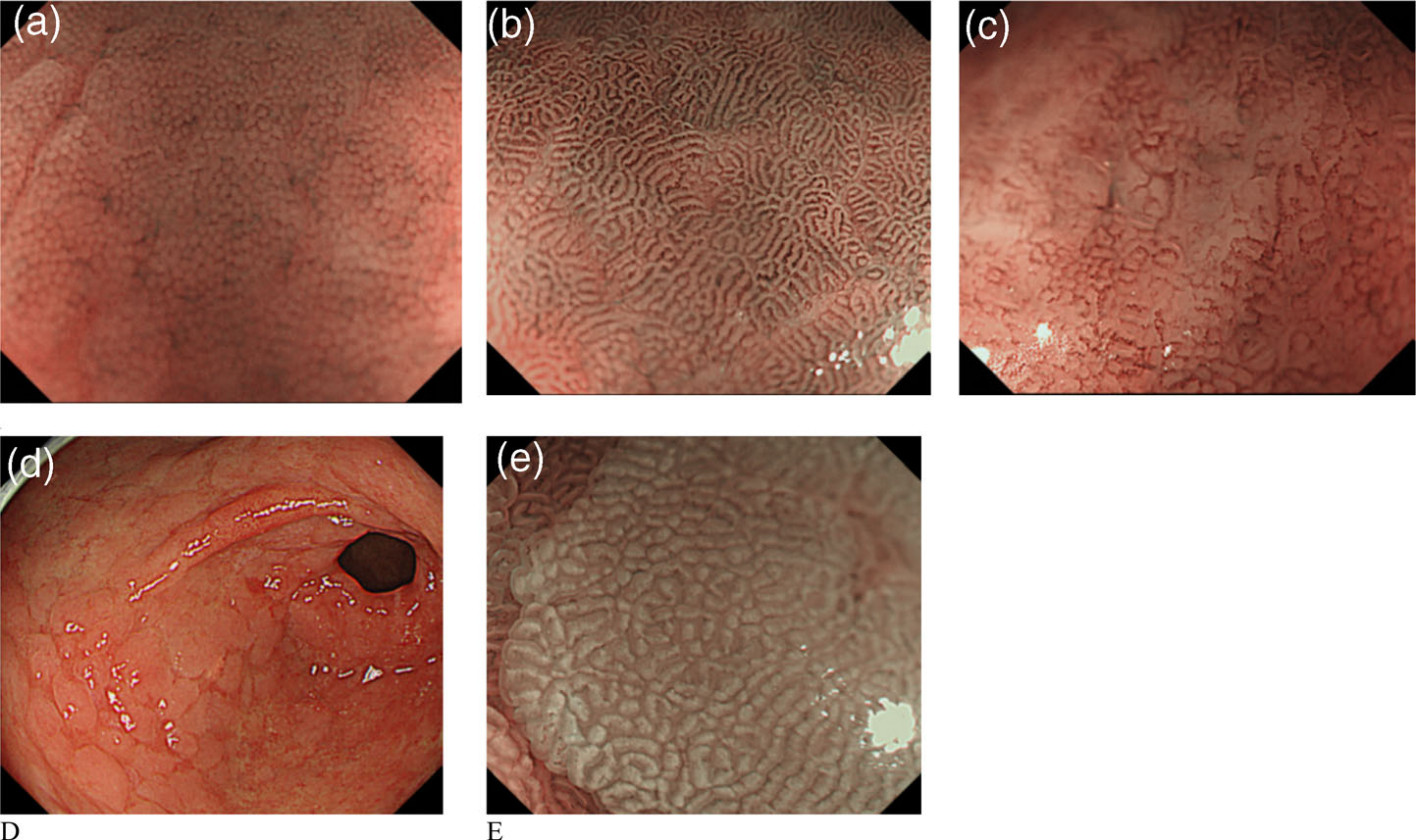
ME‐NBI finding of intestinal metaplasia featuring light blue crest. (a) White light endoscopy. (b) ME‐NBI. ME‐NBI, magnifying endoscopy with narrow‐band imaging. ME‐NBI finding in intestinal metaplasia featuring white opaque substance. (d) White light endoscopy. (e) ME‐NBI. ME‐NBI, magnifying endoscopy with narrow‐band imaging.^4^

Observation of gastric pit and vascular patterns in the gastric body with ME‐NBI is effective in chronic H. pylori‐associated gastritis. These findings include: (1) absence or disorganized collection of venules, (2) indistinct or irregular gastric pits, (3) disappearance of normal SECN (Figs. 5 and 6).

A meta‐analysis was conducted on ME‐NBI, and demonstrated H. pylori infection sensitivity and specificity of 96% and 91%, respectively.^36^ Qi et al. identified 100%, 92.7%, 83.8%, and 100%, sensitivity, specificity, PPV, and NPV of ME‐NBI, respectively.^35^ Other studies also found high sensitivity (79%) and specificity (81%) of the ME‐NBI for H. pylori infection status to the gastric mucosal appearance.^44^, ^45^

Hiromitsu Kanzaki et al. evaluated areae gastricae pattern in the corpus with 0.2% indigo carmine chromoendoscopy for the diagnosis of chronic fundic gastritis (CAFG), the extension of which was evaluated with autoflurescence imaging endoscopy, and micro‐mucosal structure was examined with magnifying chromoendoscopy and NBI. They illustrated that most areae gastricae showed a foveola‐type micro‐mucosal structure (82.7%), while the intervening part of areae gastricae had a groove‐type structure (98.0%, *P* < 0.001). Groove‐type mucosa had a higher grade of atrophy (*P* < 0.001) and intestinal metaplasia (*P* < 0.001) than with foveola type.^46^ Yasushi Yamasaki et al. also evaluated areae gastricae pattern in the antrum with 0.2% indigo carmine chromoendoscopy for the diagnosis of chronic fundic gastritis (CAFG), the extension of which was evaluated with autoflurescence imaging endoscopy and micro‐mucosal structure was examined with magnifying narrow‐band imaging (M‐NBI) and classified it into groove and white villiform types. Associations among the extent of CAFG, micro‐mucosal pattern, and histology were examined. As the extent of CAFG increased, the proportion of white villiform type mucosa increased, whereas that of groove‐type mucosa decreased (*P* = 0.022). The white villiform type mucosa had significantly higher grades of atrophy (*P* = 0.002) and intestinal metaplasia (*P* < 0.001) than groove‐type mucosa.^47^

### Magnifying endoscopy with blue light imaging (ME‐BLI)

**Figure 7:**
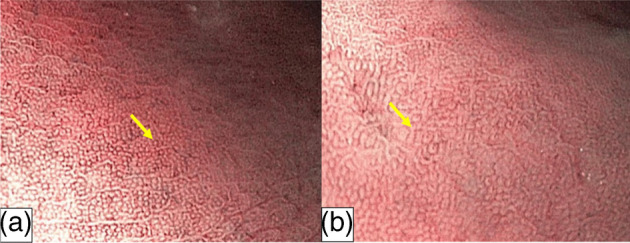
(a) H. pylori‐negative gastric mucosa is characterized by homogeneous, round pits with regular honeycomb‐like SECNs in NBI; (b) H. pylori‐positive gastric mucosa is characterized by enlarged or elongated, varies in sized and shaped of pits with unclear SECNs in NBI.^6^

Due to dark field patterns, blue light imaging technology is limited. However, blue light imaging presents clear mucosal and vascular structure appearance with high contrast laser light system. ME‐NBI and ME‐BLI are effective models for differentiation of H. pylori gastritis and have high sensitivity of 98% and specificity of 92% (see Fig. 7).^29^

### Linked color imaging (LCI)

**Figure 8:**
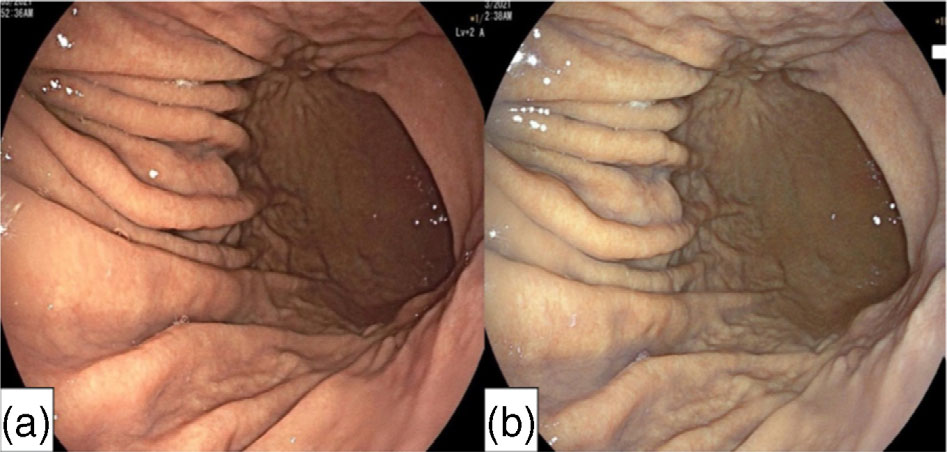
(a) H. pylori‐negative infection gastric mucosa in WLI; (b) light orange/white apricot gastric mucosa in LCI.^6^

**Figure 9:**
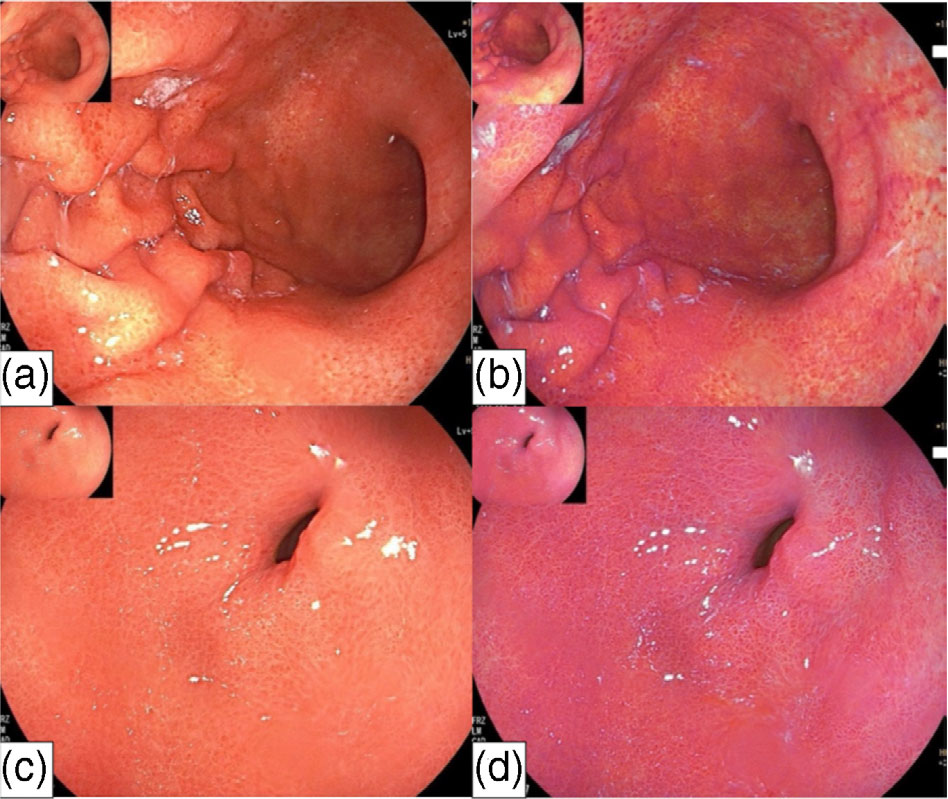
(a) Diffuse redness in gastric body in WLI; (b) Diffuse redness in gastric body (Deep Reddish Color) in LCI; (c) Diffuse redness in gastric antrum in WLI; (d) Diffuse redness in gastric antrum (Deep Red Color) in LCI.^6^

The contrast of LCI is twice higher than white light imaging. Moreover, recent studies have been reported three time higher detection rate than conventional white light imaging in the differentiating of abnormal lesions (see Figs. 8 and 9).^6^ A study in Japan identified positive H. pylori gastric mucosa by LCI more accurately with 93.8% sensitivity and 78.3% specificity than white light imaging.^11^, ^48^

### Artificial intelligence (AI)

A large prospective randomized controlled study in Japan compared AI and expert endoscopists for the accuracy of diagnosis of H. pylori infection. A total of 32 208 endoscopic images from eight sections of the stomach were classified for H. pylori positive and negative status. In the result, they identified that AI has higher sensitivity and specificity than endoscopists (81.9%, 83.4% vs. 79% 83.2%).^49^ Another experimental study in Japan compared Blue Light Image‐Bright (BL‐bright), Linked Color Imaging (LCI), and conventional white light image (CWLI) with artificial intelligence (AI), the AUC of AI‐BLI‐bright and AI‐LCI were 0.96 and 0.95, respectively, and the AUC of CWLI was 0.66.^49^ AI is considered a strong focusing method on the global health development system; therefore, studies of Artificial Intelligence endoscopic models are increasing and trying to be applied together with IEE models to improve the endoscopic diagnosis of H. pylori infection status.^50^ The advantages of AI are providing a second opinion, finding some important features during endoscopy, reducing time consumption, and requiring less training and experience. In the future, AI may become the best diagnostic method for detection of H. pylori infection status.

### Autofluorescence imaging (AFI)

**Figure 10:**
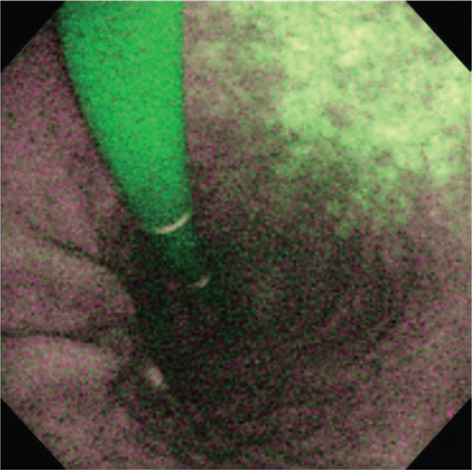
Representative case of closed‐type atrophic fundic gastritis in autofluorescence imaging (AFI) images. Cardia is surrounded by purple mucosa, and a color border between green and purple is observed at a lesser curvature of gastric body.^53^

**Figure 11:**
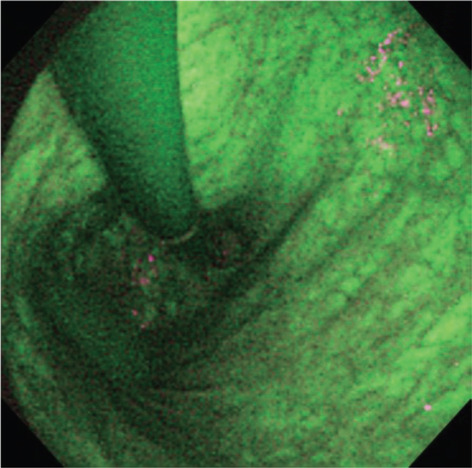
Representative case of open type atrophic fundic gastritis in autofluorescence imaging (AFI) images. The entire gastric mucosa including cardia looks bright green.^53^

Videoendoscopy of autofluorescence image (AFI) is a technique of natural tissue autofluorescence that is emitted by light excitation from endogenous fluorophores such as collagen, nicotinamide, adenine dinucleotide, flavin and porphyrins, and in the result produce real‐time pseudocolor images.^51^ Mucosal features, which are not visible with conventional white light endoscopy, AFI can enable detection of these mucosal patterns and might improve the identification and characterization of the gastritis and premalignant status in gastric mucosa.^52^ Bright green images of gastric mucosa using AFI indicate more inflammation, atrophy, or intestinal metaplasia induced by H. pylori infection. The accuracy for detecting atrophy and intestinal metaplasia was 88% and 81%, respectively, while per‐biopsy analysis, the accuracy was 76% for both. However, purple or deep green mucosal images indicate normal fundic mucosa (see Figs. 10 and 11).^52^, ^54^ Takuya Inoue et al. performed a study in 209 on the diagnosis of chronic atrophic fundal gastritis with AFI and illustrated the accuracy of green mucosa in patients with activity, inflammation, atrophy, and intestinal metaplasia of 64%, 93%, 88% and 81%, respectively; in per‐biopsy analysis, the accuracy for activity, inflammation, atrophy, and intestinal metaplasia was 55%, 62%, 76%, and 76%, respectively. Therefore, AFI may be a useful adjunct to endoscopy to identify patients at high risk of developing gastric cancer.^52^ Another study in Japan was conducted, which received eradication therapy for H. pylori infection and the extent of chronic atrophic fundic gastritis was evaluated by AFI. They revealed that metachronous early gastric cancer developed after successful H. pylori eradication, and extensive atrophic fundic gastritis, which diagnosed by AFI is a significant predictor.^53^

### EndoFaster (new innovative tool)

This is a real‐time analytic machine using gastric juice and provide information on gastric pH and ammonium concentration, which was first introduced in 2005. This machine detects the urease enzyme through a urease test in gastric juice, which is produced by H. pylori infection. A volume of 2–4 mL of gastric juice aspirate during OGD and analyzed using the EndoFaster within 1 min.^55^, ^56^ A large prospective study compared the EndoFaster and urea breath test (UBT) with histological examination as the gold standard for diagnosis of H. pylori infection identified 90.3% and85.5% sensitivity and specificity, respectively. Many other studies also reported high accuracy for the real‐time H. pylori infection diagnosis, and it is comparable with the urea breath test (UBT). The overall benefits of EndoFaster are being less invasive, not requiring proton pump inhibitor (PPI), discontinuation before testing, and less costs. Moreover, recent studies have demonstrated that EndoFaster is an useful tool for the detection of hypochlorhydric and neoplastic risk conditions, and as an adjunct to gastroesophageal reflux (GERD) treatment.^57^

### Diagnostic performance of H. pylori infection in various endoscopic models and clinical applicability

Each endoscopic model and technique could be used for demonstrating H. pylori infection status using the specific endoscopic features. Currently, the focus is on Helicobacter pylori infection, specifically on patients who are H. pylori‐positive, those who have undergone successful eradication, and individuals who have never been infected. A summary of the endoscopic features of H. pylori‐positive, eradicated and never‐infected gastric mucosa across various techniques is presented in the Tables 5, 6, 7, respectively.

**Table 5:** Summary of endoscopic features of H. pylori‐positive gastric mucosa in various models^6^, ^28^, ^29^

Endoscopic models	H. pylori‐positive gastric mucosal features		
	Endoscopic features	Sensitivity	Specificity
WLI	Diffuse redness	57.50%	95.80%
	Antral nodularity	100%	100%
	Spotty hemorrhage at fundus	61.00%	95.80%
	Enlarged gastric folds	60.10%	92.20%
	Sticky tenacious mucus	53.30%	95.10%
	Xanthoma	11.20%	98.00%
LCI	Diffuse redness (deep red color)	93.30%	78.30%
	Antral nodularity	25%	100%
	Spotty hemorrhage at fundus	50%	100%
	Enlarged gastric folds	15%	100%
	Sticky tenacious mucus	5%	100%
	Xanthoma	5%	100%
NBI	Elongated pits, variable sizes and shapes	N/A	N/A
	Obliterated collecting venules	97.00%	81.00%
BLI	Elongated pits, variable sizes and shapes	N/A	N/A
	Obliterated collecting venules	98.00%	92.00%

BLI, blue laser imaging; H. pylori, Helicobacter pylori; LCI, linked color imaging; NBI, narrow‐band imaging; WLI, white light imaging.

**Table 6:** Summary of endoscopic features of H. pylori‐eradicated gastric mucosa in various models^6^, ^28^, ^29^

Endoscopic models	H. pylori‐eradicated gastric mucosal features		
	Endoscopic features	Sensitivity	Specificity
WLI	Diffuse redness	57.50%	95.80%
	Antral nodularity	100%	100%
	Spotty hemorrhage at fundus	61.00%	95.80%
	Enlarged gastric folds	60.10%	92.20%
	Sticky tenacious mucus	53.30%	95.10%
	Xanthoma	11.20%	98.00%
LCI	Diffuse redness	93.30%	78.30%
	Antral nodularity	25%	100%
	Spotty hemorrhage at fundus	50%	100%
	Enlarged gastric folds	15%	100%
	Sticky tenacious mucus	5%	100%
	Xanthoma	5%	100%
NBI	Elongated pits, variable sizes and shapes	N/A	N/A
	Obliterated collecting venules	97.00%	81.00%
BLI	Elongated pits, variable sizes and shapes	N/A	N/A
	Obliterated collecting venules	98.00%	92.00%

**Table 7:** Summary of endoscopic features of H. pylori‐never infected gastric mucosa in various models^6^, ^28^, ^29^

Endoscopic models	H. pylori‐never infected gastric mucosa		
	Endoscopic features	Sensitivity	Specificity
WLI	RAC	92.40%	94.50%
	Fundic gland polyp	14.60%	95.50%
	Hematin spots	12.80%	93.80%
	Red streaks	100%	2.80%
	Raised erosion	2.80%	99.10%
LCI	Light orange/white apricot mucosa	96.70%	50%
	RAC	76.70%	90%
	Fundic gland polyp	13.30%	100%
	Hematin spots	16.70%	100%
	Red streaks	16.70%	100%
	Raised erosion	10%	100%
NBI	Round homogenous sized pits and presence of RAC	80%	85%
BLI	Round homogenous sized pits and presence of RAC	80%	95%

BLI, blue laser imaging; H. pylori, Helicobacter pylori; LCI, linked color imaging; N/A, not available; NBI, narrow‐band imaging; RAC, regular arrangement of collecting venules; WLI, white light imaging.

### Comparison of various endoscopic models with the H. pylori infection status

A randomized prospective comparative study used WLI, LCI, NBI, and BLI techniques simultaneously for the diagnosis of H. pylori status between 220 and 2021 was conducted at Thamsat University Hospital, Thailand. Endoscopic appearance of gastric mucosa to H. pylori infection status present as diffuse redness, especially in the fundus with spotty hemorrhage, enlarged gastric folds, and sticky mucus strongly suggest a positive H. pylori infection; the presence of regular arrangement of collecting venules (RAC) predict H. pylori non‐infected status and had a high negative predictive value (NPV). Image‐enhanced endoscopy (IEE), especially ME‐BLI model, can improve the clear visualization of the mucosa and microvascular structures, which are important to evaluate H. pylori infection status and pre‐cancerous lesions.^6^ They revealed that image‐enhanced endoscopic (IEE) models can improve the diagnostic performance of H. pylori infection status than white light image (WLI) endoscopic model. Sensitivity, specificity, PPV, NPV, and total accuracy of white light endoscopy (WLI), image enhance endoscopy (IEE), and artificial intelligence (AI) in conducted studies are summarized in Table 8. The development of endoscopic techniques can reflect histologic patterns of endoscopic images during endoscopy for the real‐time diagnosis of H. pylori infection.^58^

**Table 8:** Sensitivity, specificity, PPV, NPV, and total diagnostic accuracy of Helicobacter infection in various endoscopic models in recent studies

	Endoscopic method	Sensitivity	specificity	PPV	NPV	Total Dx accuracy for HP	
Chatrangsun and Vilaichone^6^	WLI	90.00%	70.00%	66.70%	91.30%	78.00%	
	LCI	95.00%	76.70%	73.10%	95.80%	84.00%	
	BLI	95.00%	80.00%	76.00%	96.00%	86.00%	
	NBI	85.00%	80.00%	73.90%	88.90%	82.00%	
Lee et al.^30^	WLI	32.4%	93.3%	85.2%	53.6%	70.8%	
	LCI	57.4%	91.3%	88.7%	64.3%	78.8%.	
Jiang et al.^31^	LCI	83.8%	99.5%				
Wang et al.^32^	Corpus LCI	85.41%	79.71%	59.42%	94.02%		
Ono et al.^33^						Current infection	Past infection
	WLI	84.4%	74.6%			79.5%	36.8%
	LCI	84.4%	88.9%			86.6%	78.9%
Chen et al.^34^	LCI	78.38%	70.97%	82.5%	59.46%	87.84%	
	MI	81.98%	81.25%	83.87%	64.10%	91.67%	
	M‐LCI	78.38%	80.65%	76.25%	57.78%	92.42%	
Tahara et al.^29^	NBI	97%	81%	87%	95%		
	BLI	98%	92%	93%	98%		
	M‐BLE	98%	92%				
Qi et al.^35^	M‐NBI	100%	92.7%	83.8%	100%		
Capelle et al.^36^	M‐NBI	96%	91%				
Bang et al.^37^	AI	A reliable tool for endoscopic diagnosis of H pylori infection.			Lacking external validation performance and being conducted only in Asia should be overcome		

BLI, blue light imaging; CI, confidence interval; LCI, linked color imaging; NBI, narrow‐band imaging; M‐BLE,^6^ magnifying with blue light imaging; M‐LCI, Magnifying with Linked color imaging; M‐NBI, Magnifying with narrow‐band imaging; NPV, negative predictive value; PPV, positive predictive value; WLI, white light imaging.

## Conclusion

Recent studies demonstrate that various endoscopic imaging models of the gastric mucosa improve the evaluation and detection of H. pylori status and pre‐cancerous lesion by the development of new equipment and models and can present valuable information for clinical and histopathological correlations.

Conventional white‐light imaging endoscopy with a modified scoring system of the Kyoto classification of gastritis due to its availability, widespread use, less time‐consuming, low cost, and no need for more experience is an effective model for the evaluation of H. pylori infection status and pre‐cancerous lesion of gastric mucosa. Newly developed image‐enhanced endoscopic (IEE) models alone or in combination with magnifying endoscopy are more effective models for the detection of gastric mucosal changes and obtaining accurate targeted biopsies than conventional white light imaging (CWLI) endoscopy. However, using IEE modules requires adequate training and skills along with sufficient experience, time, and specialized advanced equipment to perform the procedures. Therefore, its widespread use is limited and more studies have not been conducted. The use of AI is limited, and there are no further research studies, but it will be a good diagnostic model for the evaluation of H. pylori infection status in the future because of the detection some important features during endoscopy, performed in a short time and require less training.
